# Supplementary figures and images for: A postmeiotically bifurcated roadmap of honeybee spermatogenesis marked by phylogenetically restricted genes
Source: PLoS Genet. 2023 Dec 4;19(12):e1011081. doi: 10.1371/journal.pgen.1011081 (PMC10721206; doi:10.1371/journal.pgen.1011081)

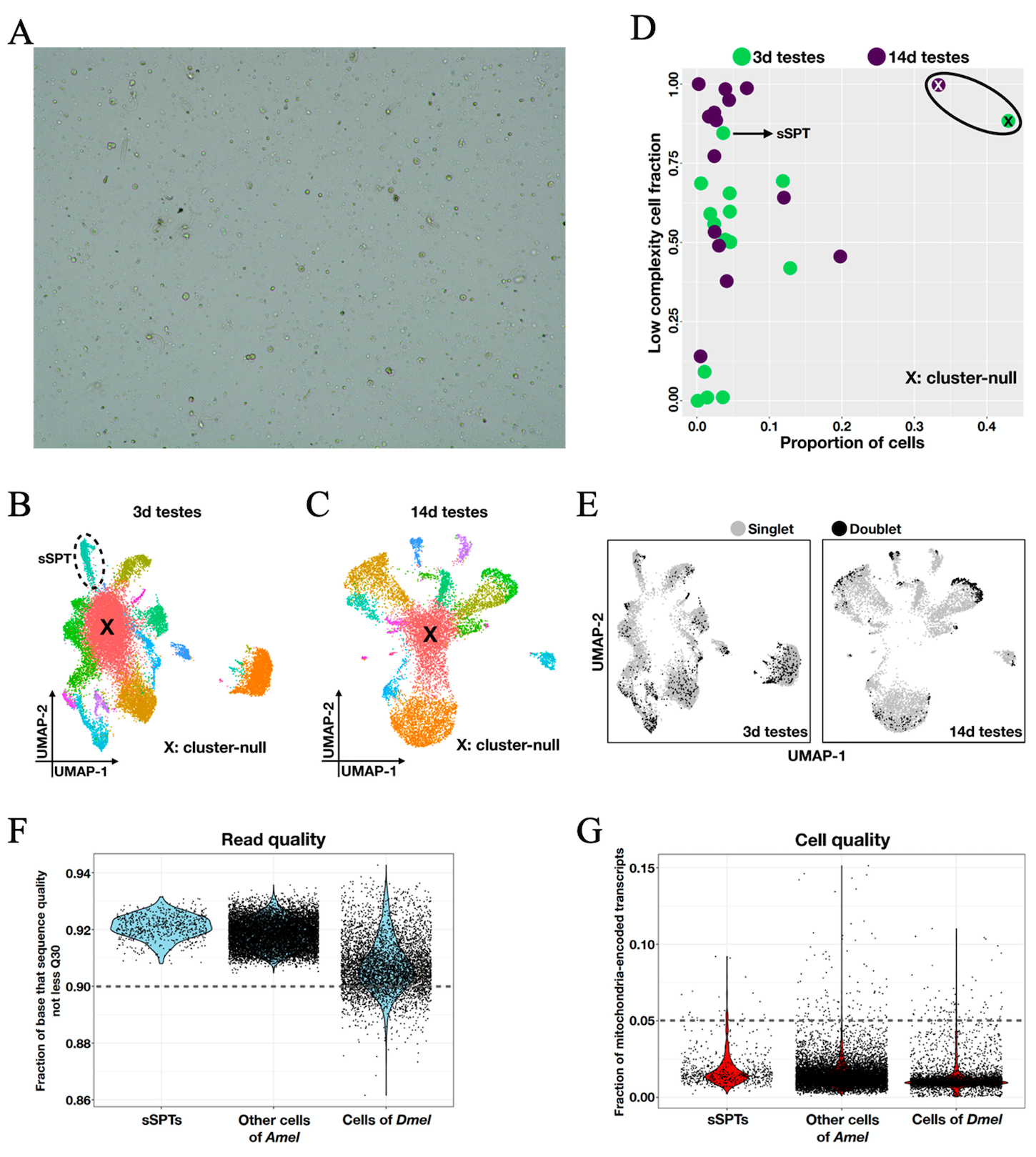

Supplement: S1 Fig — (A) A representative bright-field image of single-cell preps from the 3d adult testes. The preps were stained with acridine orange and propidium iodide. The images were captured and analyzed by Countstar Rigel S2. (B-C) UMAP projections of 20,436 and 11,097 cells from 3d (A) and 14d (B) adult testes, respectively. Seurat parameters: HVF = 1,050, PC = 13, and resolution = 0.5 (see Materials and Methods). Note that the sSPT cluster of cells (dashed circle) were clearly separated from cluster-null (red) in the 3d dataset. (D) A scatter plot of the proportion of low-complexity cells (gene number < 2,000 and UMI < 5,000) in each cluster against the share of this cluster in all cells. Green: 3d; black: 14d. Note that the sSPT cluster (arrow) was distant from clusters-null (solid circle). (E) Identified doublets from 3d and 14d dataset, respectively. (F) The average read qualities in the sSPT cell type, all the other Amel cell types and all the Dmel cell types identified from the data of [50]. (G) The single-cell qualities measured as the fraction of mitochondria-encoded transcripts. (TIF) [file pgen.1011081.s001.tif]

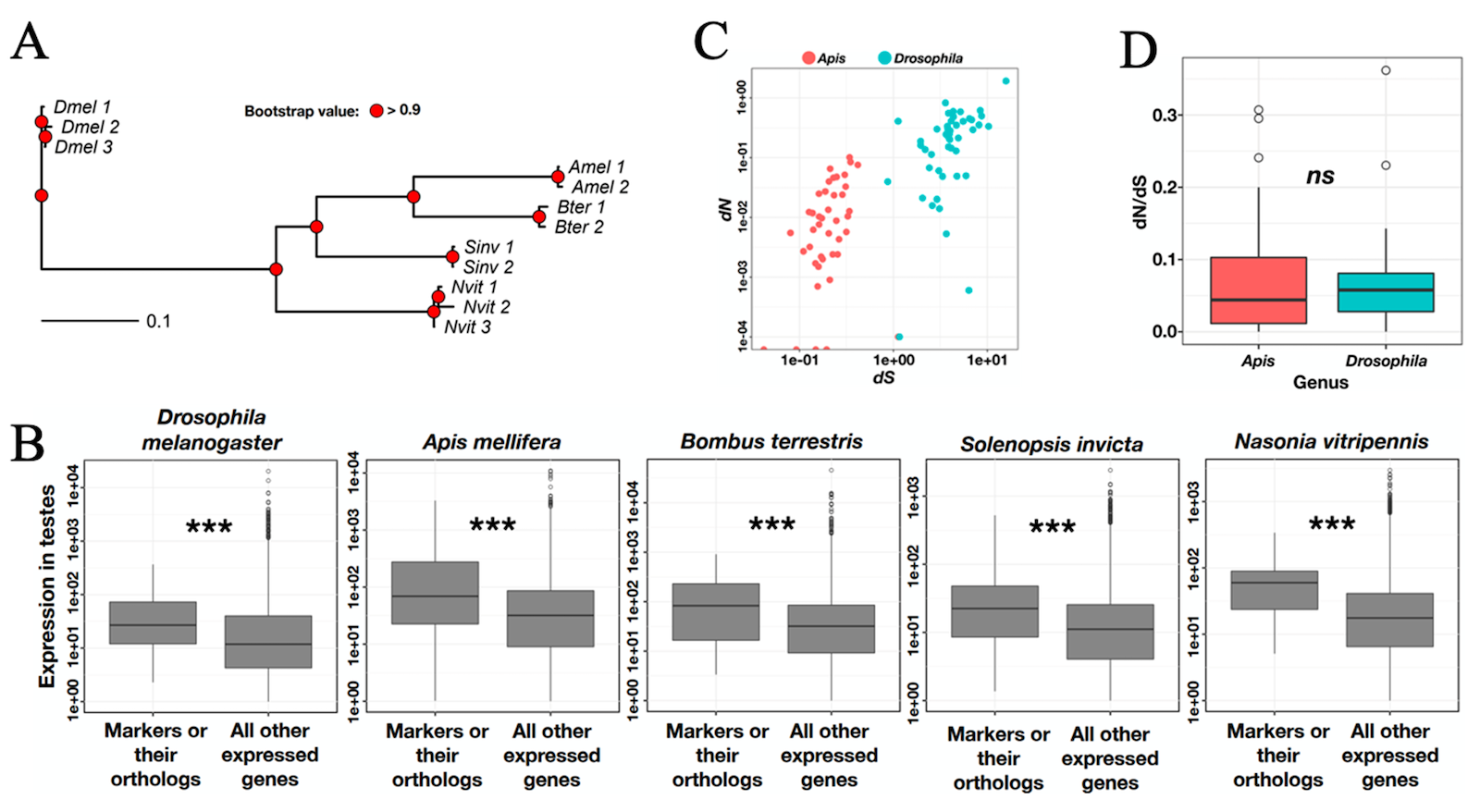

Supplement: S2 Fig — (A) Expression phylogeny of the 53 spermatogenic cell type-specific marker genes based on the bulk RNA-seq data from adult testes of five species: Drosophila melanogaster (Dmel), Apis mellifera (Amel), Bombus terrestris (Bter), Solenopsis invicta (Sinv) and Nasonia vitripennis (Nvit). Bootstrap analyses (a total of 4,974 one-to-one orthologous genes were randomly sampled with replacement for 1,000 times) suggest a high reliability of the branching pattern (proportions of replicate trees > 0.9). (B) The Amel marker genes and their counterparts in the other four species all show an overall higher level of testicular expression. *** denotes a p-value < 0.001 of Wilcoxon rank sum test comparing the marker genes with all the other genes with FPKM > 1 or RPKM > 1 in the corresponding testis bulk RNA-seq datasets (Materials and Methods). (C) The distributions of dS and dN for the spermatogenic cell type-specific marker genes shared by the genera Apis (Apis mellifera, Apis cerana, Apis dorsata and Apis laboriosa were used in the calculation; red) and Drosophila (Drosophila melanogaster, Drosophila simulans, Drosophila yakuba, Drosophila pseudoobscura and Drosophila grimshawi; green). (D) The distributions of dN/dS for the spermatogenic cell type-specific marker genes shared by Apis and Drosophila. Wilcoxon rank sum test p-value = 0.8. (TIF) [file pgen.1011081.s002.tif]

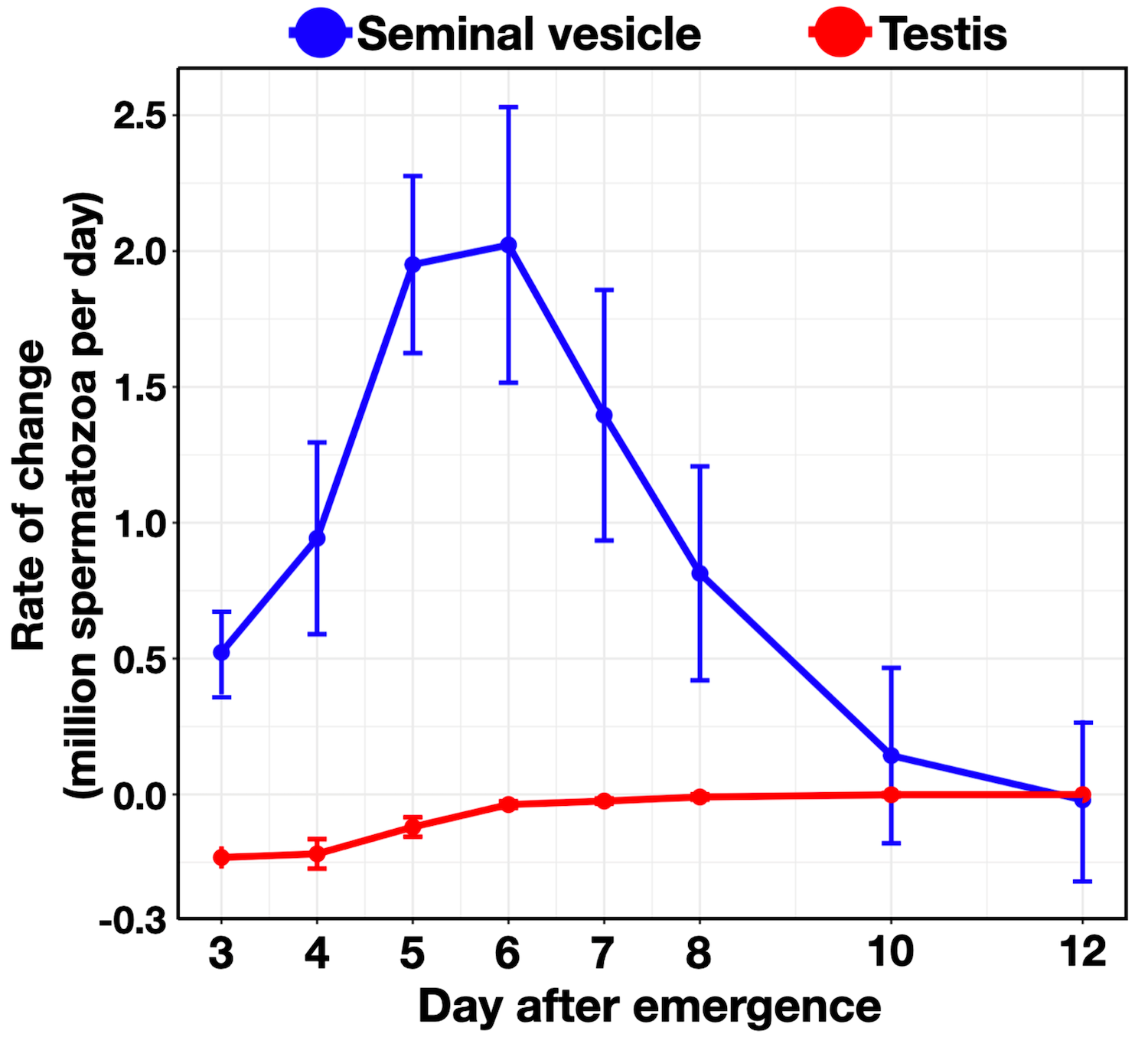

Supplement: S3 Fig — The raw data (N = 20 independent samples on each day) were shown in Fig 1C. Bootstrapping was performed by resampling the data for 20 times for each day. Then the change rate on each day was estimated as the slope of linear fit with two preceding data points, the current data point and the following two data points in each bootstrapping vector. Student’s t-tests were used to statistically compare the change rates between seminal vesicle measurements and testis measurements. (TIF) [file pgen.1011081.s003.tif]

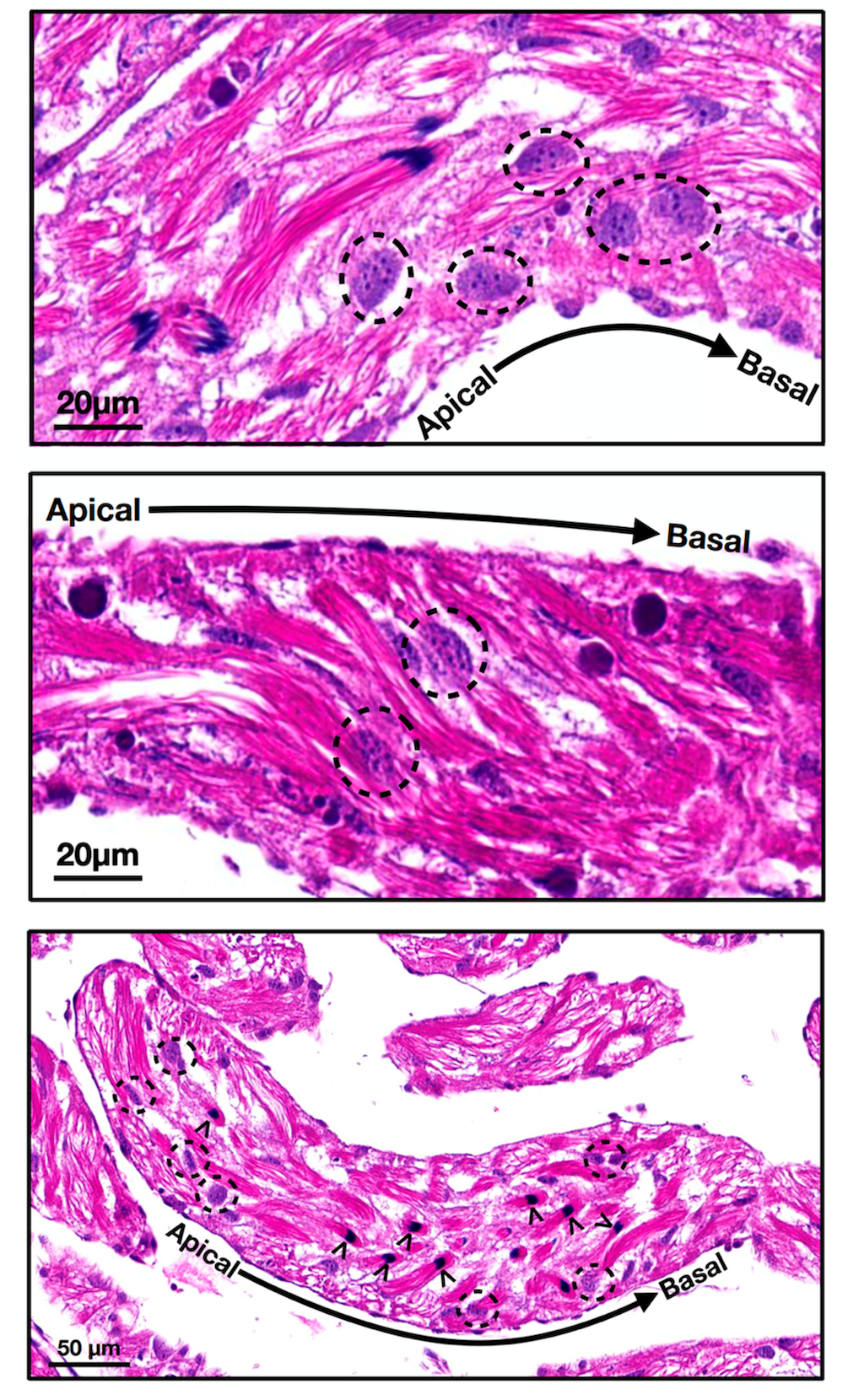

Supplement: S4 Fig — Hematoxylin & Eosin staining were used. Dashed circle: spermatogenic cyst; arrowhead: spermatid bundle. (TIF) [file pgen.1011081.s004.tif]

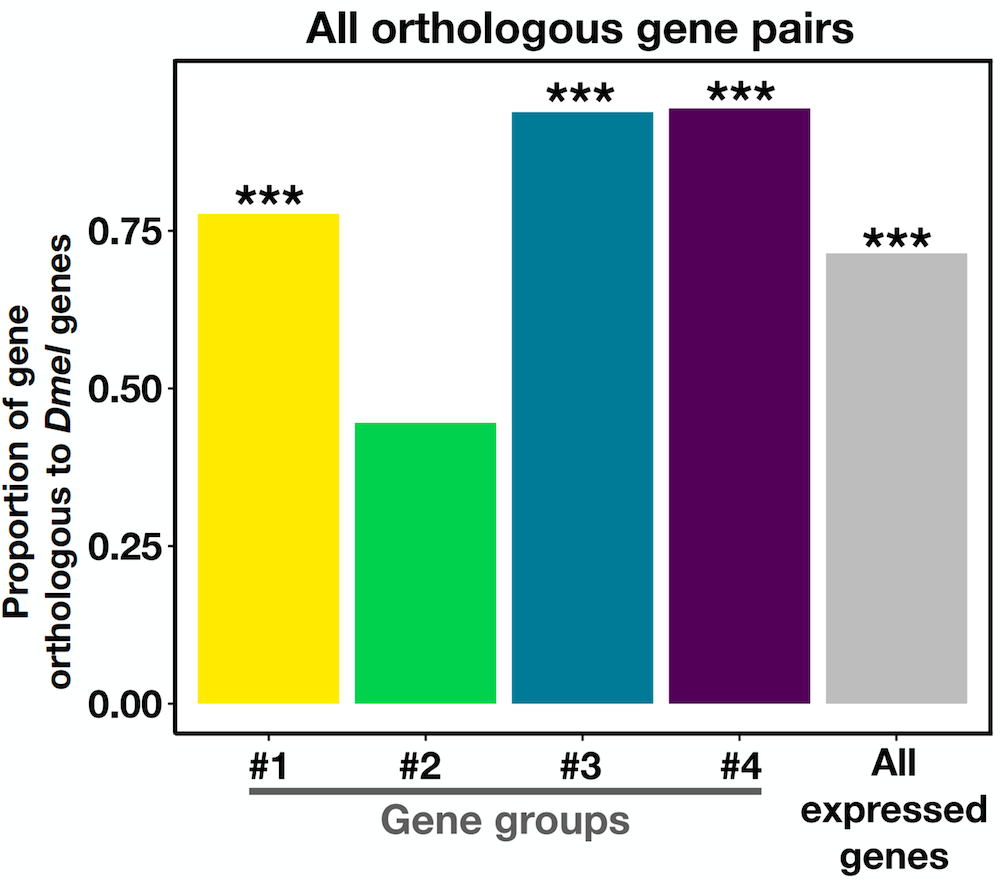

Supplement: S5 Fig — *** denotes Fisher’s exact test p-value < 0.001. (TIF) [file pgen.1011081.s005.tif]

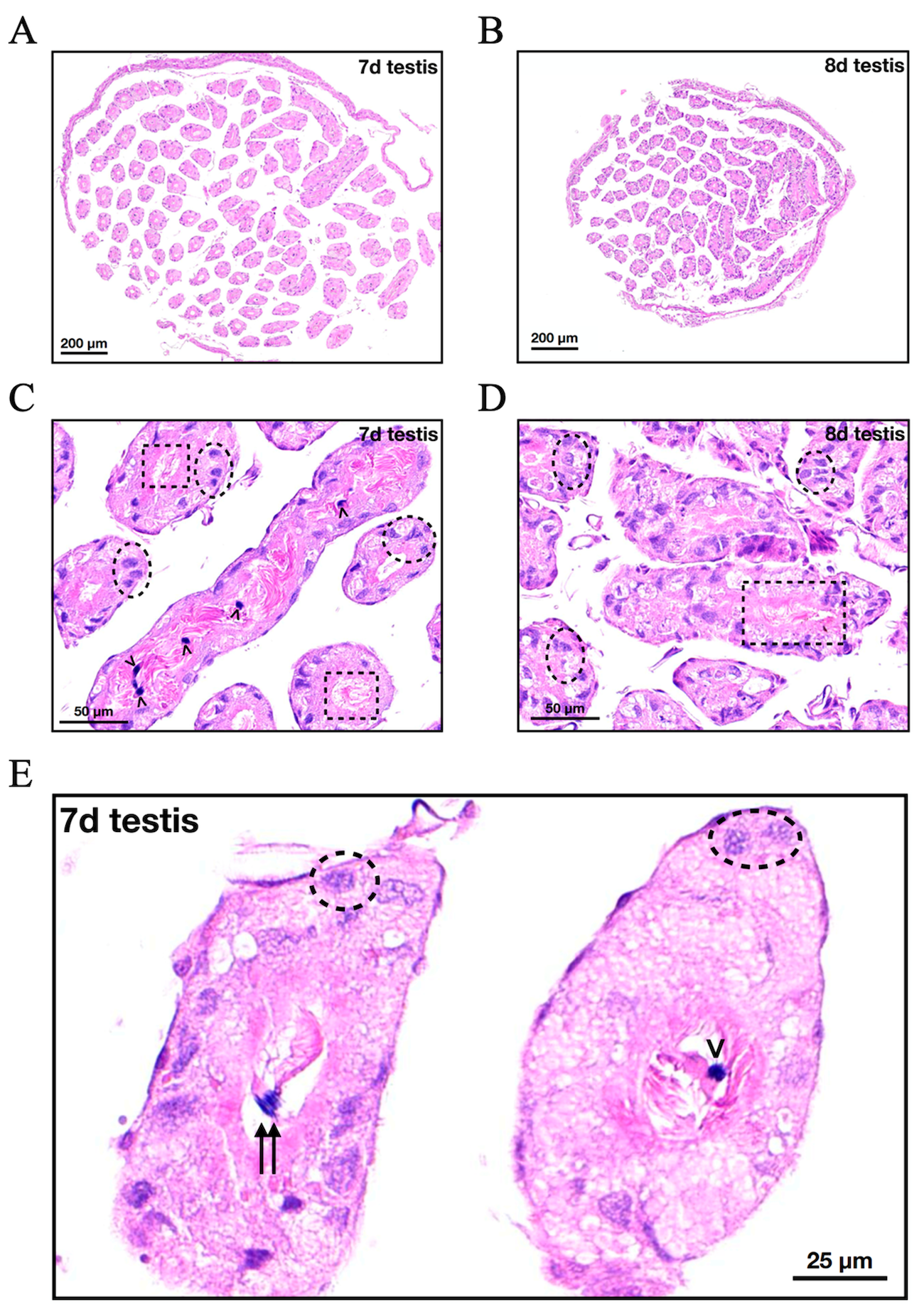

Supplement: S6 Fig — Dashed circle: spermatogenic cyst; arrowhead: spermatid bundle; double-arrow: spermatid bundle undergoing individualization; dashed rectangle: individualized spermatids. (TIF) [file pgen.1011081.s006.tif]

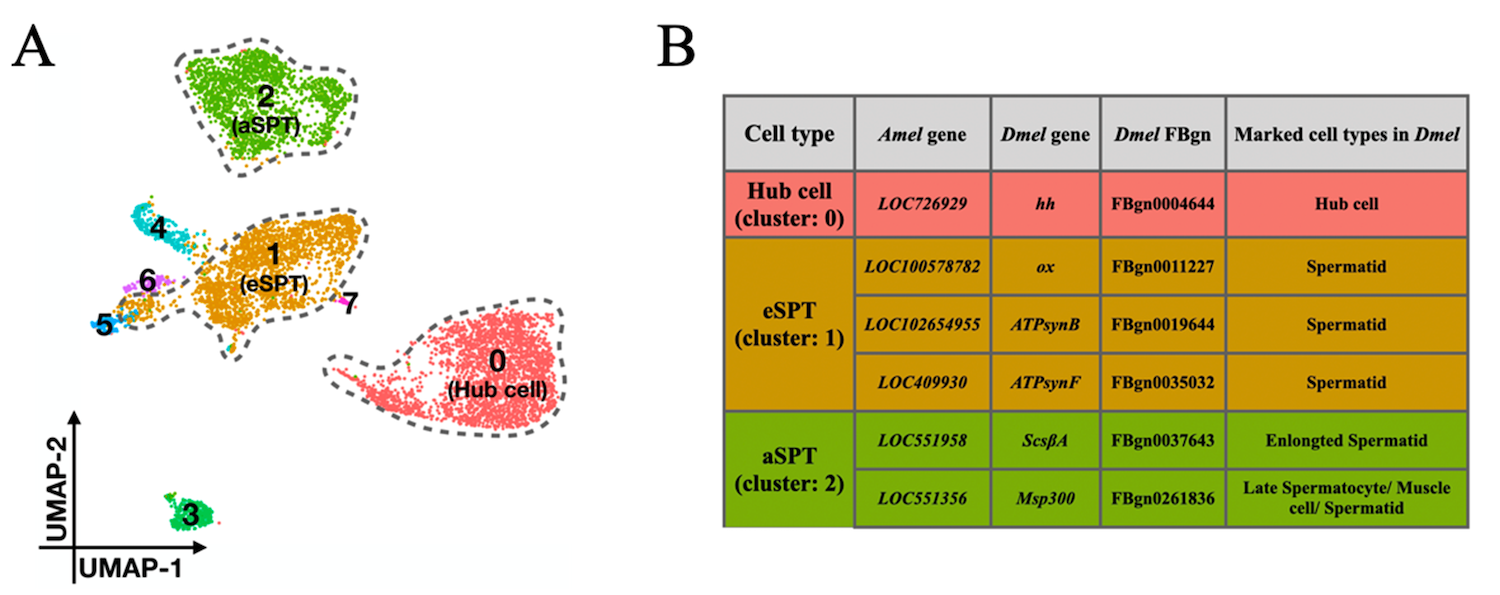

Supplement: S7 Fig — (A) One cluster of hub cells and two clusters of spermatids were identified with the 64 marker genes established in the analysis of 3d data (Fig 1F). (B) The marker genes of the three cell types identified from the 14d-only data. (TIF) [file pgen.1011081.s007.tif]
