# Supplementary material for: A postmeiotically bifurcated roadmap of honeybee spermatogenesis marked by phylogenetically restricted genes
Source: PLoS Genet. 2023 Dec 4;19(12):e1011081. doi: 10.1371/journal.pgen.1011081 (PMC10721206; doi:10.1371/journal.pgen.1011081)
Supplement: Attachment — Submitted filename: PLoSGen_review_notes 1 (002).docx [file pgen.1011081.s014.docx]

A postmeiotically bifurcated roadmap of honeybee spermatogenesis marked by phylogenetically restricted genes

**Overview:**

This manuscript provides a detailed mapping of spermatogenesis at the single-cell level between recently emerged (3d) and sexually mature (14d) honeybee drones. The authors provide a well-characterised dataset using Drosophila marker genes to annotate cell types and identify a small subset of small spermatids in the newly emerged drones. They also describe ongoing sperm production post-emergence shown by the presence of cell-types and markers characteristic of the germline stem-cell niche and increasing spermatid numbers. This work will be a useful data source to better understand spermatogenesis in the honeybee and other Hymenoptera species by providing species-specific gene markers for the stages of male gamete production.

The annotation of clusters needs to be more clearly supported e.g. a more detailed explanation of the marker genes used with citations to show the appropriateness of said genes. Additionally, figure legends need to be more detailed; for example, abbreviations explained etc.

A few minor suggestions below:

**Introduction**

**Paragraph 2**: typo ‘development of honeybee’ and ‘in fruitfly’…. missing ‘the’?

**Paragraph 3:** Most of this paragraph might be better placed in results/discussion.

**Results:**

***Testis scRNA-seq reveals a continuous process of spermatogenesis in newly-emerged honeybee drones***

**Paragraph 1:**

‘visually inspected to be healthy’ could be more clear as to examples of what might be excluded.

States that 3d and 14d old drones were dissected and proceeds to describe data collected from drones aged 1-18 days in Fig 1C.

**Paragraph 2:**

‘showed testicular expression’ – explain what this means

‘corresponding bulk RNA-seq datasets’ - where are these described?

**Paragraph 3:**

‘Among these genes, 109 had enriched expression in at least one single-cell cluster of the 3d dataset

(Figure 1E; Materials and Methods)’ – Needs more information on thresholds for defining ‘enriched’ genes, even in the methods.

‘iteratively removed genes with either inconsistency or high ambiguity’ - any particular thresholds set for removal?

Why are late spermatogonia/spermatocytes abbreviated to aSPG and aSPC?

‘LOC409668 (Dmel\PI31), LOC727432 (Dmel\I-2), LOC100577491 (Dmel\smn) and LOC414041 (Dmel\Unr)’ – References to show significance of these genes? The annotation of the clusters needs to be more clearly supported.

***The earliest spermatogonial cells express germline stem cell maintenance genes***

**Paragraph 1:**

‘However, in the daily measurements of the sperm numbers, we detected a slight increase of the testicular spermatozoa at day 2 after emergence’ – statistically significant?

‘During days 4-8, the gain rate in SVs was greater than the loss rate in testes by 0.7~2.0 million per day (Figure S2)’ – statistically significant?

**Paragraph 2:**

‘top enrichment in functional categories such as…’ – More info on gene ontology analysis either here or in methods. E.g. what exact test and correction for adjusted p-values?

**Paragraph 3:**

‘eSPG1 > eSPG4 > eSPG2 > eSPG0 > eSPG3’ – rename according to order?

‘known GSC markers in Dmel, including nanos, LOC409301 (Dmel\Mad), LOC409713 (Dmel\hts), LOC412256 (Dmel\E(bx)), LOC725878 (Dmel\how), LOC727235 (Dmel\ena) and LOC100576993 (Dmel\zfh1)’ – References?

***Spermatogenesis in honeybee is characterized by a branch point after meiosis II***

**Paragraph 1:**

‘the aSPC-SPT branch appeared to uniquely represent a meiotic cell fate in honeybee’ – Typo? aSPC-sSPT?

‘Both groups showed a dynamic expression pattern with a peak level at the branch point, suggesting an active event of meiotic division II at this time (Figure 3B)’ – peak not particularly clear.. especially for G2/M genes. Not sure that the data fully supports this claim.

***Hymenoptera-restricted genes are highly expressed in small spermatids***

**Paragraph 1:**

‘the unique sSPT branch may be reflective of transcriptional programming events led by phylogeny specific genes in honeybee’ – what is the logic behind this hypothesis? Citations for context?

‘we examined the orthology relationship between Amel and Dmel in genes with sSPT-high expression (Group #2 in Figure 3D). We found that 44.5% of Group #2 genes share orthology with Dmel genes, and the proportion is significantly lower than any other group of genes’ – How does one conclude this from figure 3D? Should reference 4B here?

***Transcription regulatory network uncovers transcription factors that contribute to small spermatid specification***

**Paragraph 1:**

‘from our full-length RNA-seq data, scored motif clusters of annotated transcription factors for each TSS, and selected regulatory edges with significant weights and top-scored target genes to build the final regulons for SPTs and sSPTs (Materials and Methods)’ – not clear what exactly was done here, what was the scoring system?

How was the ‘connectivity’ mentioned in Figure 5C measured?

**Paragraph 2:**

‘In the sSPT network, 5 out of the 139 active TFs were significantly enriched HRGs as the relevant targets’ – Unclear, rephrase.

‘and sSPT-specific expression (Figure 5F)’ – Foxp and LOC100576369 also show significant increase in sSPT?

***Spermatogenesis is largely absent in 14-day-old adult honeybee testes***

**Paragraph 2:**

‘A riverplot shows the assignments of each integration cluster to the two datasets, revealing a lack of early spermatogenic processes and an accumulation of late spermatid cells in 14d testes (Figure 6E)’ – Confusing plot. 14d only data has not been introduced?

‘This finding was further verified by the bulk mRNA-seq detection of a general decrease in the maker genes for early spermatogenic cell types, including eSPG, aSPG, eSPC and aSPC (Figure 6F)’ – This plot shows 3d vs 15d when 3d vs 14d is what was being discussed prior.

***Mutation load is decreased in spermatids of 14-day-old adult honeybee testes***

**Paragraph 1:**

Typo: ‘3d and 14 testes’

‘By contrast, sSPTs had a higher per-base rate than either eSPTs or aSPTs, and this difference was greater than twofold’ – giving exact difference would be better.

**Figures:**

**Figure 1D:** May be clearer to label clusters with inferred cell type for consistency with text and panels 1G-H

**Figure 4:** Account for multiple testing with adjusted p-values?

**Figure S3:** Define arrow heads etc in legend.
