# Supplementary material for: A postmeiotically bifurcated roadmap of honeybee spermatogenesis marked by phylogenetically restricted genes
Source: PLoS Genet. 2023 Dec 4;19(12):e1011081. doi: 10.1371/journal.pgen.1011081 (PMC10721206; doi:10.1371/journal.pgen.1011081)
Supplement: Attachment — Submitted filename: Response to reviewers.docx [file pgen.1011081.s015.docx]

>>Reviewer #1: In this manuscript, Yi and colleagues analyze spermatogenesis of the honeybee with single-cell sequencing. Honeybees are a fascinating and understudied species, with an unusual meiotic program: haploid males, produced through asexual reproduction, fail to undergo a reduction division in meiosis to retain a haploid state in their gametes. Some data, as cited by the authors, suggest that honeybee male meiosis II is asymmetric, as reminiscent of mammalian female meiosis: each meiotic II division gives rise to one spermatid and one small haploid cell. To study this interesting phenomenon, the authors perform single-cell RNA sequencing on honeybee testes. Because the honeybee is so understudied relative to its potential, this dataset is of great interest to germ cell biologists generally. The dataset appears to generally be of high quality, and should absolutely be published (and I think that PLOS Genetics is an undeniably appropriate journal for such a dataset to be published in). That being said, I have some concerns about the validity of the cell type annotation, which is necessary to build the subsequent conclusions of the paper.

Author response: We would like to thank this reviewer for his/her appreciation of the significance and quality of our work.

>>First, the authors use 1-to-1 homology with Drosophila genes to annotate their clusters. It is unclear where the Dmel cell type analysis comes from, since the reference column in the supplemental table doesn’t appear to have a corresponding reference list (or perhaps I simply couldn’t find it). Regardless, as identified by the authors, honeybee spermatogenesis occurs very differently from Dmel, both in regards to meiotic behavior and with a lack of a single, maintained stem cell population that allows for homeostatic spermatogenic turnover throughout the fly’s lifetime. Therefore, without validation of these markers in the honeybee (through RNA in situ hybridization or other methods), it’s hard to know if the assumptions that Dmel factors have the same cell type expression is appropriate. Likewise, there’s also some mixed information about these markers: zfh1, for example, is listed as a GSC marker in the text, but as a cyst cell marker in the supplementary table.

Author response: We thank this reviewer for raising this important issue. As a well-studied model organism, *Dmel* also represents the phylogenetically closest species to *Amel* offering a wealth of molecular knowledge about spermatogenesis. We made use of such knowledge by curating the relevant FlyBase data and previous publications, compiling information on a set of 248 *Dmel* genes, each of which had been reported to express in specific testicular cell types (see Table S1 for references cited). A final set of marker genes used in our current work consisted of 64 orthologous genes, whose cell type-specific expressions in *Amel* were consistent with those in *Dmel* (Figure 1F). We agree with the reviewer’s concern that experimentally validated marker genes in *Amel* would have been ideal particularly when comparing two species that exhibit differences in details in spermatogenesis. Based on gene orthology, gene expression specificity and the new substitution rate analysis (Figure S2), we postulate that most of the marker genes selected for in our analysis likely share similar functions with their *Dmel* counterparts. Importantly, progression of cell type changes revealed in our pseudotime analysis was consistent with the sequence of the core, conserved events of spermatogenesis, supportive of the utility of the selected marker genes in our study. As further detailed below, we are cognizant that a lack of experimental validation is a limitation of our current study and have toned down our statement whenever necessary (see also our response to Reviewer #2, below).

In response to this reviewer's suggestion, we have now provided a detailed explanation of one representative marker gene for each of the spermatogenic cell types, including their expression patterns in *Amel*, the functional roles of their *Dmel* counterparts, and the *Dmel* references. This information will be useful for guiding experimental validations in future studies of spermatogenesis of the honeybee and other Hymenopteran species. In addition, we have also checked all the information about the marker genes through the manuscript. We have confirmed that *zfh1* was incorrectly listed as a GSC marker and have corrected this error.

>>Second, the most important conclusion of the paper comes from annotation of a cluster that lacks expression of Dmel orthologs, and is also generally UMI low. This cluster is annotated as “small spermatids,” or sSPTs. Notably, the authors themselves remove cells in the dataset with inconsistency or high ambiguity, relative to the Drosophila data, as well as removing low-complexity cells. It is easy to believe that the sSPT-annotated cells are simply low-quality or poorly sequenced cells, accounting for their low map rate and apparent high mutational rate. Alternatively, since the authors performed single-cell sequencing, rather than single-nuclei sequencing, it is very possible that this cell cluster arose from fragmenting spermatid heads from spermatid tails. In Drosophila, these cell types are very fragile, and cell suspensions can often contain fragments of spermatid tails that can be captured by droplet-based sequencing and mistaken for cells, since they do contain significant amounts of RNA. This could result in what appear to be two spermatid populations, when, in fact, it’s simply two parts of the spermatid sequenced separately. Again, without validation, it’s hard to know if this cell type is real or artifactual. Since many of the main conclusions of the paper depend on this annotation, it’s hard to asses them.

Author response: We thank this reviewer’s evaluation, and we indeed also entertained the possibility that small spermatids (sSPTs) identified in our dataset might simply be spermatid fragments or cytoplasm blebs. Spermatids could be fragmented in the experimental procedures due to their fragility as suggested by this reviewer. Blebs of cytoplasm could result from either cytoplasmic extrusion during spermiogenesis or cytoplasmic division during the aborted meiosis I in the honeybee. These cellular fragments and blebs might have been captured by the droplet-based approach to contribute to the low-complexity single-cell cluster (Figure S1). However, our evidence argues strongly against this possibility. First, both the read quality and the single cell quality in the sSPT cluster were as high as the other testicular cell types in our honeybee data and in published fruitfly datasets (Figure S1E-F). Second, in the unsupervised clustering analysis, the presence of sSPT cluster was robust and largely independent of the parameter choice (nFeature cutoff, resolution for principal component analysis and the number of clusters). Third, the sSPT cluster was clearly separated from the low-complexity cluster on UMAP (Figure S1). Fourth, the transcriptomic features of sSPTs were biased toward germ cell types rather than somatic cell types (Figure 1H). In particular, the sSPT cluster was correlated the best with late spermatocytes (Pearson’s *R* = 0.94), favoring the hypothesis of sSPTs being derived from late spermatocytes instead of spermatids. Importantly, we were compelled by the evidence that cells in the identified sSPT cluster were uniquely characterized by an enriched expression of Hymenoptera-restricted genes with their expression levels reaching or surpassing those of other genes. This result makes it very difficult to imagine, virtually ruling out the possibility that the identified sSPTs were merely cytoplasmic fragments derived from other cells. To enhance the presentation of our work, we have now included these discussions in the revised manuscript.

>>Overall, I recommend this paper for publication with significant revisions. The data is worthy and should be published, but the conclusions are overstated with the level of analysis done. Either the authors should validate their cell type annotations in vivo using their newly identified honeybee marker transcripts, or they should clearly lay out the limitations to the analysis given the potential issues with annotation.

Author response: We once again thank this reviewer for his/her support and appreciation of our work. The honeybee is a finicky organism that is grossly understudied. While our current work has been limited by the accessibility of healthy drones for accurately determining the age and collecting samples, it represents the first single-cell transcriptomic study of spermatogenesis in the honeybee. Due to technical limitations, we have now toned down our conclusions whenever appropriate, and we have provided a separate paragraph in Discussion to specifically lay out such limitations.

>>Reviewer #2: This study utilized single-cell RNA sequencing to examine spermatogenesis in honeybee drones at two developmental timepoints, 3 days and 14 days after emergence. The authors identified distinct spermatogenic cell populations in 3-day-old drones, including a small spermatid population potentially arising from asymmetric meiotic division. They found active spermatogenesis in 3-day drones but not in 14-day drones, indicating spermatogenesis is largely complete by 14 days of age. The experiments appear technically sound, but there are some concerns about the biological interpretations that require further clarification. The authors relied heavily on known markers from Drosophila to identify cell types. However, meiosis I and testis morphology differ substantially between Drosophila and Apis. To strengthen the conclusions, the authors should perform comparative analyses using genetic data from other hymenopteran species. This may allow better identification of honeybee-specific cell types based on evolutionary signatures rather than assuming orthology with Drosophila.

Author response: We thank this reviewer for his/her appreciation of the soundness of our data and analysis. We understand this reviewer’s concern about the use of marker genes from *Drosophila* in our analysis given the well appreciated morphological differences of testes between the two species. As detailed in our response to Reviewer #1, the marker genes selected for our analysis are expected to exert biological functions conserved between these two species to support the conserved (invariable and maybe fundamental) events of spermatogenesis. While this notion remains to be validated experimentally in future studies, we have taken this reviewer’s advice in performing an informatics analysis across different species. Here, we collected adult testis bulk RNA-seq data of *Dmel*, *Amel*, and three other Hymenopteran species: *Bombus terrestris* (*Bter*), *Nasonia* *vitripennis* (*Nvit*) and *Solenopsis* *invicta* (*Sinv*). We found that the transcriptional divergence of these marker genes followed the known Hymenopteran phylogeny, suggesting that the expression changes of these genes are steadily accumulated within Hymenoptera (Figure S2A). We confirmed that these marker genes were expressed at relatively high levels in the transcriptome of adult testes in all these five species (Figure S2B). Furthermore, we calculated synonymous substitution rates (*d*_S_), nonsynonymous substitution rates (*d*_N_) and *d*_N_/*d*_S_ ratios of these genes across the genus of *Drosophila* or *Apis*. We found that while both *d*_S_ and *d*_N_ were significantly smaller in *Apis*, in consistence with the phenotypical difference between the two genera (Figure S2C), no significant difference was detected in *d*_N_/*d*_S_ (Figure S2D). This result suggests that the spermatogenic marker genes are constrained by strong purifying selection in both *Apis* and *Drosophila*, further supportive of their functional conservation. We have included a new paragraph in the main text to discuss these results and implications.

>>The small spermatid population is an intriguing finding. To definitively demonstrate their origin and fate, functional experiments tracking these cells over development would significantly enhance this study. Their transient presence and high mutation load suggest they may contribute to sperm quality control, but it is unclear why honeybees uniquely produce small spermatids. Further studies of their functional roles across more timepoints is needed. In summary, this study provides valuable single-cell transcriptomic resources for honeybee spermatogenesis. However, some conclusions require more functional validation and evolutionary comparisons between hymenopteran species. Addressing these concerns would significantly strengthen this contribution.

Author response: We thank this reviewer for recognizing the value of our dataset and significance of identifying small spermatids (sSPTs) in the honeybee. We also understand the concerns raised by this reviewer (and Reviewer #1) with regard to experimental validation and have accordingly toned down many of our conclusions (see also our response to Reviewer #1). We would like to note that, as suggested by this reviewer (see above), we have performed an informatics analysis as an alternative--though imperfect--to experimental validation in functional investigation into the biology that we study. We wish to draw the reviewer’s attention to the fact that our work is the first study using single-cell transcriptome approach to a long-standing question in biology. While important questions remain that require future studies (e.g., why the bees uniquely produce small spermatids as noted by this reviewer), we hope that the reviewer and editor would agree that our current study, given its well-defined scope and carefully toned conclusions that are reflective of the technical limitations, is a unit of scientific contribution sufficient for publication.

>>Additional specific issues were raised:
Figure 1: The authors chose 3d and 14d drones to prepare scRNA-seq libraries from freshly dissected testes. Then identified different cell types in the testicular tissue of the 3d drones, and suggesting a continuous process of spermatogenesis presented in the newly emerged honeybee testes. The article points out that 3d of age represents a transition stage when a massive number of spermatozoa are transferred from testes to seminal vesicles. However, from the results of Figure 1B, it seems that a 4-day drone is more appropriate. Please explain it.

Author response: We agree with the reviewer that the transferring rate of spermatozoa from testes to seminal vesicles in the 3d drones was lower than the 4d drones. In fact, we chose 3d in our analysis for two major reasons. 1) We suspected that the testes at the first two days after emergence could be insufficiently mature thus resembling too closely to those in the pharate-adult stage. 2) We aimed to examine whether the major spermatogenic cell types were present in 3d that represented the onset of the transition stage. We have revised the relevant statement in the main text to improve the clarity of the presentation of our work.

>>Figure 2: The identification of putative germline stem cell markers in early spermatogonia is an interesting finding. Further functional validation could strengthen the conclusion that these cells have differentiation potential.

Author response: We thank this reviewer for his/her appreciation of our finding about putative germline stem cell (GSC) markers in the honeybee. This information will be useful in future efforts in identifying and locating male GSCs during the testicular development of the honeybee and other Hymenopteran species, studies that we believe would be outside the scope of the current work.

>>Figure 3: The authors note that pseudotime trajectories for meiotic cells typically show a continuous developmental progression in mouse and fly studies. However, they identified a unique bifurcation in honeybees, with a branch giving rise to aSPC-sSPT cells. This aSPC-sSPT branch appears to represent a distinct meiotic cell fate in honeybees. Initial identification of these cell populations relied on known marker genes from Drosophila due to conserved aspects of spermatogenesis. However, the emergence of small spermatids from asymmetric meiotic division seems to be a more lineage-specific feature in bees. The authors could perhaps comment in more detail on both the similarities to Drosophila meiosis based on orthologous markers, as well as any bee-specific characteristics of the transcriptomic profiles and regulatory relationships uncovered in the aSPC-sSPT branch. This may provide better evolutionary context for interpreting this key result.

Author response: We thank the reviewer for giving us an opportunity to further elaborate on the observed branch and its relation with *Amel*-specific and *Dmel*-*Amel* shared features. As noted in our response to this reviewer (above) and Reviewer #1, our current results are supportive of the notion that orthologous marker genes with known spermatogenic roles in *Dmel* also function in supporting the conserved events of meiosis and spermatogenesis in *Amel*. Consistent with this idea, the orthologous genes with known roles during *Dmel* meiosis II exhibited a dynamic expression pattern along the pseudotime axis from SPCs to SPTs/sSPTs in *Amel* (Figure 3B up). Among these genes, *LOC727405* showed the clearest expression peak at the branch point, resembling the dynamic pattern of its *Dmel* ortholog, *mud*, around meiosis II (Figure 3B down). The *mud* gene encodes an essential component of the meiosis II spindle apparatus in oocytes, and interestingly, its proteins are prominently present at the center of polar bodies (Yu et al. 2006). The *mud* gene is also expressed in the *Dmel* spermatozoa, but this functional role is unknown (Wasbrough et al. 2010). Thus, *LOC727405*/*mud* represents an orthologous gene functioning in the conserved events of meiosis. This conserved aspects of meiosis and spermatogenesis were further supported by gene ontology analysis (Figure 3C). The transcriptomic features of SPCs, SPC-to-SPT/sSPT transition and SPTs were in general consistence with the knowledge in *Dmel* (Figure 3D). On the contrary, the sSPT-specific features did not match the known features of any spermatogenic cell types of *Dmel*. For example, the newly identified SPC-sSPT branch showed an enrichment in the gene ontology of cilium. This feature is common in late-stage spermatids of *Dmel*, but it is paradoxically more pronounced in sSPTs than in SPTs of *Amel*. In addition, an analysis without relying on orthology revealed that sSPTs were in fact characterized by Hymenoptera-restricted genes (HRGs), further supporting that the new branch is unique to *Amel*. Thus, our results suggest that the constellation of sSPT-specific features provides the molecular basis for the specification of the unique small spermatids in *Amel*.

>>Figure 4: The authors showed that transcriptional activities of HRGs and pre-HRGs were comparable in spermatids, which differs from their expression patterns in whole-body and whole-testis samples. It would be helpful for the authors to also examine HRG/pre-HRG expression differences specifically among the highly expressed genes in the whole-body and whole-testis data. Adding this analysis would allow for a more equivalent comparison to the spermatid expression data, where only the highly expressed HRG/pre-HRG subsets were analyzed. Looking at the HRG/pre-HRG expression ratios among highly expressed genes across all sample types could provide a clearer picture of any tissue and cell-type specific biases.

Author response: We thank this reviewer for this constructive suggestion. We have now replaced Figure 4D-E with the analysis of HRG/pre-HRG expression differences among the highly expressed genes. The analysis is indeed a more equivalent comparison to the sSPT data in Figure 4F, and our conclusion remains the same.

>>Figure 5: The authors should double check the statistical analysis in 5B. Validating the significance of HRG enrichment in sSPTs versus SPTs is important.

Author response: We have double checked the statistical tests throughout the manuscript. Figure 5B shows the number of network edges inferred from SPTs and sSPTs, respectively. We confirmed that the HRG-associated edges in sSPTs were moderately more (6.4% in sSPTs vs. 5.6% in SPTs; Fisher’s exact test *p* = 0.08). This result is in general agreement with the analysis of highly expressed HRG/pre-HRG shown in Figure 4C.

>>Figure 6: The authors compared the morphology of drone testicular tissue and the distribution of different sperm cell species between 3d and 14d, revealing a lack of early spermatogenic processes and an accumulation of late spermatid cells in 14d testes. However, sSPT cells did not exist in the testicular tissue at 14 days, and we could not observe the development track of sSPT cells. It is better to select the testicular tissue of drones in the intermediate period (example: 7d) for analysis, so that the comparison with 3d data can more effectively observe the changes and functions that may be involved in sSPT cells.

Author response: We agree with this reviewer in that it would be insightful to determine the drone age when meiosis II is completely aborted and when sSPTs are eliminated. Ideally, daily collections between 3d and 14d would be best in achieving such a goal, but this would be neither cost-effective nor practical at the current time. In this study, we chose 3d and 14d to test specifically against the hypothesis that spermatogenesis was fully concluded in adult drones, leading to an important conclusion which opens more questions that we would investigate in future studies. As a compromise, we have generated H&E staining images of testicular sections from 7d and 8d drones. We show that while the spermatogenic cysts were still present at this stage, the majority of bundled spermatids have become individualized (Figure S6). Thus, although spermatid individualization was seen before emergence in a previous reports (Lago et al. 2020; Klein et al. 2021), 7-8 days after emergence may be the stage when individualization massively occurs. These results further support the finding in 3d data about the ongoing spermatogenesis in adult drones. We have included these results in the main text.

>>Figure 7: The authors chose 3d and 14d drones to prepare scRNA-seq libraries from freshly dissected testes. Then identified different cell types in the testicular tissue of the 3d drones, and suggesting a continuous process of spermatogenesis presented in the newly emerged honeybee testes. The article points out that 3d of age represents a transition stage when a massive number of spermatozoa are transferred from testes to seminal vesicles. However, from the results of Figure 1B, it seems that a 4-day drone is more appropriate.

Author response: We thank again this reviewer for pointing out this issue. The inappropriate statement has now been revised. Please see our response to the comment on Figure 1.

>>Minor:
* Results p9: change ' aSPC-SPT ' for 'the aSPC-sSPT branch'
* Figure 6F: The "15 d testes" in the illustration does not correspond to the figure legends.

Author response: We thank this reviewer for pointing out these errors. They have been corrected in the revised manuscript.

>>Reviewer #3: 
Overview:
This manuscript provides a detailed mapping of spermatogenesis at the single-cell level between recently emerged (3d) and sexually mature (14d) honeybee drones. The authors provide a well-characterised dataset using Drosophila marker genes to annotate cell types and identify a small subset of small spermatids in the newly emerged drones. They also describe ongoing sperm production post-emergence shown by the presence of cell-types and markers characteristic of the germline stem-cell niche and increasing spermatid numbers. This work will be a useful data source to better understand spermatogenesis in the honeybee and other Hymenoptera species by providing species-specific gene markers for the stages of male gamete production. The annotation of clusters needs to be more clearly supported e.g. a more detailed explanation of the marker genes used with citations to show the appropriateness of said genes. Additionally, figure legends need to be more detailed; for example, abbreviations explained etc.

Author response: We thank this reviewer for his/her appreciation of our work and constructive suggestions that have improved our paper.

>>A few minor suggestions below:
Introduction
Paragraph 2: typo ‘development of honeybee’ and ‘in fruitfly’…. missing ‘the’?
Paragraph 3: Most of this paragraph might be better placed in results/discussion.

Author response: We have made these changes as suggested by this reviewer.

>>Results:
Testis scRNA-seq reveals a continuous process of spermatogenesis in newly-emerged honeybee drones
Paragraph 1:
‘visually inspected to be healthy’ could be more clear as to examples of what might be excluded.

Author response: The information about the traits we inspected for healthy drone collection has been added in the methods.

States that 3d and 14d old drones were dissected and proceeds to describe data collected from drones aged 1-18 days in Fig 1C.

Author response: We have added this statement in the main text.

>>Paragraph 2:
‘showed testicular expression’ – explain what this means

‘corresponding bulk RNA-seq datasets’ - where are these described?

Author response: We have updated this paragraph with more details.

>>Paragraph 3:
‘Among these genes, 109 had enriched expression in at least one single-cell cluster of the 3d dataset
(Figure 1E; Materials and Methods)’ – Needs more information on thresholds for defining ‘enriched’ genes, even in the methods.

Author response: We filtered the *Amel* genes sharing one-to-one orthology with known *Dmel* markers with two criteria. 1) A candidate marker gene was required to be expressed in >10% cells of at least one cluster (i.e., marked cluster). 2) The marked cluster was further required to express the candidate marker gene at a level >50% of the maximum level among all clusters. We have now included this information in both Figure 1E legend and the methods.

>> ‘iteratively removed genes with either inconsistency or high ambiguity’ - any particular thresholds set for removal?

Author response: For the 109 candidate marker genes, we assumed that a sufficient number, but not all, of them could correctly annotate the *Amel* cell types. We further filter these genes by evaluating their performances in the final annotation. For a good marker gene, if it marked only one *Amel* cell type, their correspondence must be supported by the *Dmel* knowledge in Table S1. If it marked two or more *Amel* cell types, the correspondences not supported by the *Dmel* knowledge must not exceed two. Finally, we obtained a set of 64 *Amel* genes that could sufficiently identify a total of 9 cell types in the 3d dataset. We have now included this information in the methods.

>>Why are late spermatogonia/spermatocytes abbreviated to aSPG and aSPC?

Author response: First, we tried to avoid any confusion that might be introduced by using letter “l” to stand for “late”. Second, we thought that the aSPG and aSPC cells were just developmentally later or advanced than the eSPG and eSPC cells, respectively. Therefore, we used aSPG and aSPC as the abbreviations. We have made this information clearer in the main text and Figure 1F legend.

>> ‘LOC409668 (Dmel\PI31), LOC727432 (Dmel\I-2), LOC100577491 (Dmel\smn) and LOC414041 (Dmel\Unr)’ – References to show significance of these genes? The annotation of the clusters needs to be more clearly supported.

Author response: We have now added the references wherever appropriate in the manuscript. A complete list of *Dmel* references can be found in Table S1. In addition, we included one new paragraph with detailed information about one representative marker gene for each of the honeybee spermatogenic cell type.

>>The earliest spermatogonial cells express germline stem cell maintenance genes
Paragraph 1:
‘However, in the daily measurements of the sperm numbers, we detected a slight increase of the testicular spermatozoa at day 2 after emergence’ – statistically significant?

Author response: This increase was weak. We have changed the statement to “In the daily measurements of the sperm numbers, we detected a non-significant increase of the testicular spermatozoa at day 2 after emergence (from 0.9 ± 0.2 million to 1.0 ± 0.2 million; Student’s t-test *p* = 0.23; Figure 1C red), indicative of presumed new sperm formation.”

>> ‘During days 4-8, the gain rate in SVs was greater than the loss rate in testes by 0.7~2.0 million per day (Figure S2)’ – statistically significant?

Author response: To estimate the statistical significance, we have now performed bootstrapping to obtain the distributions of the change rates on each day. The result suggests that the gain rates in SVs were significantly greater than the loss rates in testes (Student’s t-test *p*-values < 10^-7^). We have now added this information to the legend of Figure S3 and the related text.

>>Paragraph 2:
‘top enrichment in functional categories such as…’ – More info on gene ontology analysis either here or in methods. E.g. what exact test and correction for adjusted p-values?

Author response: In this study, we used the tool of HymenopteraMine to perform all *Amel* GO analysis. Hypergeometric test and Holm-Bonferroni correction were used. We have now included this information at this place of the main text and in the methods.

>>Paragraph 3
‘eSPG1 > eSPG4 > eSPG2 > eSPG0 > eSPG3’ – rename according to order?

Author response: We have renamed these cell clusters as suggested by this reviewer.

‘known GSC markers in Dmel, including nanos, LOC409301 (Dmel\Mad), LOC409713 (Dmel\hts), LOC412256 (Dmel\E(bx)), LOC725878 (Dmel\how), LOC727235 (Dmel\ena) and LOC100576993 (Dmel\zfh1)’ – References?

Author response: We have now added the *Dmel* references about these GSC marker genes.

>>Spermatogenesis in honeybee is characterized by a branch point after meiosis II
Paragraph 1:
‘the aSPC-SPT branch appeared to uniquely represent a meiotic cell fate in honeybee’ – Typo? ASPC-sSPT?

Author response: We have now corrected this typo.

>> ‘Both groups showed a dynamic expression pattern with a peak level at the branch point, suggesting an active event of meiotic division II at this time (Figure 3B)’ – peak not particularly clear.. especially for G2/M genes. Not sure that the data fully supports this claim.

Author response: We thank this reviewer for pointing out this issue. We have now rephrased the description about the dynamic pattern of meiosis II markers (Figure 3B up) to “These genes had a relatively high level of expression at the branch point…” We have removed the result of G2/M markers, which was weak and might be irrelevant. We have also provided the information about the most representative gene, *LOC727405*. This gene showed a clear expression peak at the branch point (Figure 3B down), resembling the dynamic pattern of its *Dmel* ortholog, *mud*, around meiosis II (Figure 3B inset). The *mud* gene encodes an essential component of the meiosis II spindle apparatus in the *Dmel* oocytes, and interestingly, its proteins are prominently present at the center of female polar bodies. The *mud* gene is also expressed in the *Dmel* spermatozoa, but this functional role is unknown. Thus, *LOC727405*/*mud* may represent an orthologous gene that functions in the conserved events of meiosis.

>>Hymenoptera-restricted genes are highly expressed in small spermatids
Paragraph 1:
‘the unique sSPT branch may be reflective of transcriptional programming events led by phylogeny specific genes in honeybee’ – what is the logic behind this hypothesis? Citations for context?

Author response: We have rephrased this statement to “During the gene ontology analysis (see above), we observed that a number of genes with enriched expression in sSPTs (i.e., Group #2) could not be annotated by HymenopteraMine (Materials and Methods). We suspected a contribution of non-conserved genes to the transcriptomic features of sSPTs. To test this hypothesis, we examined the orthology between *Amel* and *Dmel* for genes in Group #2.”

>> ‘we examined the orthology relationship between Amel and Dmel in genes with sSPT-high expression (Group #2 in Figure 3D). We found that 44.5% of Group #2 genes share orthology with Dmel genes, and the proportion is significantly lower than any other group of genes’ – How does one conclude this from figure 3D? Should reference 4B here?

Author response: The information about Group #2 was in Figure 3D. To avoid confusion, we have now removed “in Figure 3D” (see the above response). In addition, we have generated a new figure to show the proportions of *Amel*-*Dmel* orthologous genes in each groups (Figure S5).

>>Transcription regulatory network uncovers transcription factors that contribute to small spermatid specification
Paragraph 1:
‘from our full-length RNA-seq data, scored motif clusters of annotated transcription factors for each TSS, and selected regulatory edges with significant weights and top-scored target genes to build the final regulons for SPTs and sSPTs (Materials and Methods)’ – not clear what exactly was done here, what was the scoring system?

Author response: We used Cluster-buster (Frith 2003) to find putative target genes of every transcription factor (TF). We also used this algorithm to assign a log-likelihood score to each TF-target pair. We have now rewritten this statement with more details in the main text.

>>How was the ‘connectivity’ mentioned in Figure 5C measured?

Author response: The “connectivity” is a quantification of how well a given gene is connected within a correlation-based gene network (Warner et al. 2019). It is calculated as the sum of the sixth-powered coefficients of Pearson’s correlation between a gene and all the other genes. This information is now added in Figure 5C legend and in the methods.

>>Paragraph 2:
‘In the sSPT network, 5 out of the 139 active TFs were significantly enriched HRGs as the relevant targets’ – Unclear, rephrase.

Author response: This sentence has a typo. It should be “enriched with HRGs”. We have rephrased this sentence to: “In the sSPT network, 5 out of the 139 active TFs had target genes with relatively high HRG/pre-HRG ratios (outside of the 95% confidence interval; Figure 5D).”

>>‘and sSPT-specific expression (Figure 5F)’ – Foxp and LOC100576369 also show significant increase in sSPT?

Author response: We have double checked the statistical tests through the manuscript. In Figure 5F, both *Foxp* and *LOC100577909* showed significantly higher expression in sSPTs than in SPTs (Student’s t-test *p* = 10^-7^ and 10^-35^, respectively). The results were also confirmed by a non-parametric test (Wilcoxon rank sum test *p*-value = 10^-8^ and 10^-39^, respectively).

>>Spermatogenesis is largely absent in 14-day-old adult honeybee testes
Paragraph 2:
‘A riverplot shows the assignments of each integration cluster to the two datasets, revealing a lack of early spermatogenic processes and an accumulation of late spermatid cells in 14d testes (Figure 6E)’–Confusing plot. 14d only data has not been introduced.

Author response: Unsupervised clustering is less powerful to properly resolve cell types when the number of divergent cell types is low. In fact, it was difficult for us to annotate the cell clusters of the 14d-only data. Using the marker genes for the 3d data, we identified two SPT clusters and one somatic cell clusters, consisting of 6,020 single cells (Figure S7). We have now added this information before the integration analysis.

The integration algorithm allowed us to use the cell types identified from the 3d data to annotate the 14d data. The results further confirmed the missing early-stage cell types in 14d testes. We used the riverplot in Figure 6E to illustrate the distribution of two datasets in each integrated cell cluster.

>>‘This finding was further verified by the bulk mRNA-seq detection of a general decrease in the maker genes for early spermatogenic cell types, including eSPG, aSPG, eSPC and aSPC (Figure 6F)’ – This plot shows 3d vs 15d when 3d vs 14d is what was being discussed prior.

Author response: We have now corrected this typo.

>>Mutation load is decreased in spermatids of 14-day-old adult honeybee testes
Paragraph 1:
Typo: ‘3d and 14 testes’

Author response: We have now corrected this typo.

>>‘By contrast, sSPTs had a higher per-base rate than either eSPTs or aSPTs, and this difference was greater than twofold’ – giving exact difference would be better.

Author response: sSPTs had a higher per-base rate than either eSPTs or aSPTs by 2.9 and 3.7 fold, respectively. This information has now been given in the manuscript.

>>Figures:
Figure 1D: May be clearer to label clusters with inferred cell type for consistency with text and panels 1G-H

Author response: We used Figure 1D to show the pattern of unsupervised clustering before functional annotation. We have now added the cluster IDs next to the corresponding cell types in panels 1G-H.

>>Figure 4: Account for multiple testing with adjusted p-values?

Author response: We have now updated the results with FDR-adjusted p-values. The conclusion remains the same.

>>Figure S3: Define arrow heads etc in legend.

Author response: We have added this information in the legend.
