# Supplementary material for: A postmeiotically bifurcated roadmap of honeybee spermatogenesis marked by phylogenetically restricted genes
Source: PLoS Genet. 2023 Dec 4;19(12):e1011081. doi: 10.1371/journal.pgen.1011081 (PMC10721206; doi:10.1371/journal.pgen.1011081)
Supplement: Attachment — Submitted filename: response revision 2.docx [file pgen.1011081.s016.docx]

We thank the editorial team and all the three reviewers and for the valuable comments and suggestions. The following is our response to the two remaining issues raised by reviewer #1. We hope that you will find our response satisfactory and our work acceptable for publication in PLoS Genetics.

1) Reviewer #1: The authors responded to my first critique by adding additional analyses and language changes. However, I still have some concerns. It is very possible, as the authors state in their reply, that Dmel and Amel orthologs share similar functions. However, that does not mean that their expression is restricted to the same cell types or states. Even within the Drosophila genus, orthologs can be expressed in different cell types/states in different species. Indeed, it’s commonly accepted that since evolution more frequently acts on regulatory regions than coding regions, orthologs can have the same or similar coding region (as demonstrated by the authors in their substitution analysis), but dissimilar expression regulation. For example, pigment differences between Drosophila species are often not due to changes in Yellow or Ebony genes themselves, but changes in the regulation of those genes that results in differential cell type expression (e.g., PMID 12372246). Therefore, it’s still very difficult to determine if the annotation, based on 1-to-1 orthology between Amel and Dmel, is valid: these orthologus genes could be expressed in different cell types despite their orthology. Nevertheless, the authors refer to the annotation of the spermatogenic cell types as “unambiguous” in the text. I would posit that without validation there is, in fact, considerable ambiguity regarding their annotation.

Author response: We agree with the reviewer in that a pair of orthologous genes may have differential expression preferences in their respective species. In fact, our analysis was designed specifically to get around this issue. We assumed that despite molecular differences between species, the core or "mainstream" cellular events that mark the progression of spermatogenesis are expected to be highly conserved. Working under this framework, we selected a final set of 64 orthologous genes for our analysis that successfully gave rise to consistent cell type annotations between *Amel* and *Dmel*. This final list contained fewer than half of the initial 135 one-to-one orthologous genes suitable for testicular cell typing when considering only *Dmel*. This finding reinforces the reviewer's notion and supports our analytical approach: over half of orthologous marker genes from *Dmel* are indeed not suitable for cell typing in *Amel*. Despite our best efforts, we agree with the reviewer in that our annotations may still have an unknown level of ambiguity and imprecision, and we apologize for our previous main text that did not fully acknowledge this uncertainty. As detailed below in the list of modifications to the manuscript, we have now completely removed any expression that may be counter to the existence of this uncertainty, including “unambiguous” and “a clear sign” (see also below).

2) Reviewer #1: In response to my second critique, the authors are very convincing as to why “sSPT” annotated cells are not simply low-quality or poorly sequenced cells, as the read quality and single cell against the second potential confounding factor – that the cell cluster arose from fragmenting spermatid heads and tails. Both spermatid heads and tails individually can 1) have high and complex read counts, as both contain considerable inherited RNA, 2) robustly cluster separately, as the cells’ fragile break point at the neck is consistent, and 3) appear in pseudotime analysis as germline cells that are derived from spermatocytes, since RNAs in both the head and the tail are largely transcribed in spermatocytes. I agree that the enriched expression of Hymenoptera-restricted genes in “sSPTs” is curious, but this result alone does not rule out alternative explanations for this cluster. Perhaps the authors could show a Nomarski image of their dissociated cell prep with quantification of fragmented spermatids. Additionally, such an image would allow for observation and quantification of morphologically sSPTs vs. SPTs in their cell preps. If the rate of fragmentation is very low, and the presence and ratios of (morphologically) sSPTs vs. SPTs tracks with the number of cells present in those annotated clusters, I agree it is likely that the annotated sSPTs are a bona fide cell state. As it stands, though, a limitations paragraph at the end does not change the confident language used throughout the paper to refer to this ambiguous cluster, including a sentence just one paragraph prior stating that “Our data captures a unique type of cells, small spermatids, which exhibit a clear sign of arising from an uneven meiosis II”. I do not believe that that there is such a clear sign, nor that the dataset has obviously captured these cells.

Author response: We thank this reviewer for appreciating the existence of a cluster of cells that we refer to as sSPTs reflective of their unique features such as an enriched expression of Hymenoptera-restricted genes. We agree with the reviewer in that a definitive designation of these cells as sSPTs will require studies that go beyond the transcriptomic analysis presented in the current work. Accordingly, we have eliminated any expression that may be construed as a definitive documentation of such a cell type in our analysis (see also above). As suggested by this reviewer, we have made an attempt to determine whether it was possible to distinguish these sSPT cells and cellular fragments in the bright-field images of single-cell preps for generating our data. While we did observe some fragmented spermatids, the variety among the testicular cells in the preps made the identification of sSPTs unattainable (Figure S1A). Definitively identifying these cells will require future studies, including the use of sSPT marker genes in combination of morphological analysis at the time when they are born of the spermatocyte cysts. Despite these limitations, we would like to note that, as appreciated by all the reviewers, our work stands as the first effort of using single-cell transcriptome approach toward a long-standing question regarding the asymmetric meiosis II during spermatogenesis of the bee species.

All the key points raised by the reviewers (including the suggestions made by reviewer #3 in the attached file during the first revision) and portions of our responses have been appropriately incorporated in the manuscript. The changes that we have made during this revision are listed below.

1) On line 17 page 2, “cells progressing toward spermatids (SPT) and small spermatids (sSPT)” is changed to “cells progressing toward the annotated spermatids (SPT) and small spermatids (sSPT)”.
2) In Figure S1A, a representative image of the single-cell prep shows the variety in the testicular cells of the 3d honeybee drones.
3) On line 148 page 5, “unambiguous” is removed.
4) On line 300 page 9, “a few Hymenoptera-restricted genes exhibit a clear sign of specific upregulation” is changed to “a few Hymenoptera-restricted genes exhibit a notable sign of specific upregulation”.
5) On line 362 page 11, “sSPTs might be eliminated” is changed to “sSPTs likely have been eliminated”.
6) On line 412 page 12, “greatly” is removed.
7) On line 413 page 12, “a clear sign of arising from an uneven meiosis II” is changed to “properties consistent with an origin from an uneven meiosis II”.
8) One lines 426-437 page 13, the language in the last paragraph of Discussion is polished.
